# Editorial: Exploiting Novel Combined Host- and Pathogen-Directed Therapies for Combating Bacterial Multidrug Resistance

**DOI:** 10.3389/fimmu.2020.616486

**Published:** 2020-11-04

**Authors:** Roberto Nisini, Marco R. Oggioni, Gian Maria Rossolini, Maurizio Fraziano

**Affiliations:** ^1^ Department of Infectious Diseases, Istituto Superiore di Sanità, Roma, Italy; ^2^ Department of Genetics and Genome Biology, University of Leicester, Leicester, United Kingdom; ^3^ Dipartimento di Farmacia e Biotecnologie, Università di Bologna, Bologna, Italy; ^4^ Clinical Microbiology and Virology Unit, Careggi University Hospital, Florence, Italy; ^5^ Department of Biology, University of Rome Tor Vergata, Rome, Italy

**Keywords:** multidrug resistance, bacteria, host-directed therapy, pathogen-directed therapy, innate immunity

The golden age of antibiotic therapy started in 1928 with the discovery of penicillin and reached a peak at the mid-1950s. Thereafter, antibiotic discovery and development of new molecules gradually declined with the parallel emergence of drug resistance of many human bacterial pathogens. These circumstances led to the current therapeutical crisis due to antimicrobial resistance (1). Today, the frequency and spectrum of antibiotic resistance in specific bacterial pathogens continues to increase worryingly, with particular concerns on *Mycobacterium tuberculosis* and on several Gram-positive (e.g., *Streptococcus pneumoniae*, *Staphylococcus aureus*, and enterococci) as well as Gram-negative bacteria (e.g., *Klebsiella pneumoniae*, *Escherichia coli*, Enterobacter spp, *Acinetobacter baumannii*, and *Pseudomonas aeruginosa*). The slow-pace of discovery of novel antimicrobial agents, the dearth of new antibiotics already in the drug development pipeline, and the emergence and rapid diffusion of strains resistant to last resort antibiotics, make novel therapeutic approaches an urgent need to reduce the burden of infectious diseases. It is estimated that deaths due to antibiotic resistant bacterial pathogens may pass from the actual 700,000 cases to about 10 million per year by 2050, if adequate countermeasures are not undertaken.

Novel antimicrobials or antimicrobial combinations may help to overcome this global emergence. Zhou et al. report data showing that the combined use of the antibiotics colistin and tigecycline may represent a valuable therapeutic option against multi-drug resistant *E. coli* harbouring bla_NDM-5_ and mcr-1 expression. Yong et al. show that berberine hydrochloride, a commonly used traditional Chinese medicine with known antimicrobial effects, in combination with fluconazole, may be an effective therapeutic option for infections related to FLC-resistant *C. albicans*. Sadgrove and Jones highlight the importance of pharmacokinetic and pharmacodynamic analysis in the field of ethnopharmacology, before extrapolating enteral and topical therapeutic value of natural compounds.

However, the evolution of bacteria towards resistance to antimicrobial agents, including multidrug resistance, is an unavoidable phenomenon because it reflects an aspect of the general evolution of bacteria which is unstoppable (2) and, for many bacterial infections, drug resistant mutants are likely present by the time antibiotic treatment starts. Nevertheless, such infections can be successfully cleared and it is commonly assumed that this is due to the combined action of the drug and of the immune response, the latter facilitating clearance of the resistant bacterial population (3). Novel anti-infectious therapeutic approaches based on the modulation of host response (Host-directed therapy, HDT) have been proposed to counteract the emergence of antimicrobial resistance. HDT is defined as a therapeutic approach based on strategies aimed at improving innate or adaptive protective response needed for pathogen control and/or at limiting immunopathology. In this context, the vaccination may be considered as a prototypical host-directed approach that counteracts antibiotic resistance and prevents bacterial diseases (4). HDT may also comprise any drug that can activate effector mechanisms of the antimicrobial response (ROS generation, autophagy, phagolysosome maturation, antimicrobial peptide production) and/or down-modulate tissue-damaging immune responses (5).

In this special topic, Arora et al. identify a nitroso containing pyrazolo derivative compound, which was directly effective against *M. tuberculosis*, and show a synergistic effect with isoniazid and an additive effect with other molecules. Interestingly, this molecule is also capable of inducing autophagy in host cells and this mechanism is demonstrated as the major mechanism for killing of intracellular slow- and fast- growing mycobacteria. Palucci et al. identify host trasglutaminase 2 as a possible gene target for novel host directed therapy and its inhibition by cystamine or cysteamine promotes intracellular killing of *M. tuberculosis*, and acts synergistically with a second-line anti-TB drug amikacin. Improvement in HDT strategies may also require studies focusing on the identification of microbial gene products, which could be targeted by immune responses. Thus, Santajit et al. generate a fully human single-chain variable fragment (HuscFvs) binding to N-(3-oxododecanoyl)-l-homoserine lactone (3O-C12-HSL) of *P. aeruginosa*, a quorum sensing signalling molecule that contributes to the pathogenesis of infection by regulating expression of bacterial virulence factors causing intense inflammation and toxicity in the infected host. In this study, HuscFvs is capable of neutralizing 3O-C12 -HSL activity and preventing host cell apoptosis. Finally, Ihara et al. demonstrate that dense granule proteins 7, 14 and 15 from type II *Toxoplasma gondii* strains induced host immunity *via* NF-kB activation and can limit parasite expansion.

The emergence of antimicrobial resistant strains is often caused by an inefficient immune response, which promotes the persistence of naturally occurring MDR strains within a bacterial population. Thus, patients with defective immune responses and that are unresponsive to standard antibiotic treatments are often characterized by a chronic tissue damaging inflammatory response. In the present collection, Watson et al. suggest a focused host-directed therapeutic approach capable of enhancing pauci-inflammatory microbial killing in myeloid phagocytes, which maximizes pathogen clearance while minimizing the harmful consequences of the inflammatory responses. The combined down-modulation of the pathogenic inflammatory response and activation of the antimicrobial response has been described by Poerio et al. The authors show that the treatment with apoptotic body-like liposomes loaded with phosphatidylinositol 5-phosphate promotes phagosome maturation, which is naturally subverted in cells from CF patients, and intracellular bacterial killing of MDR *P. aeruginosa*, while simultaneously limiting inflammatory response both *in vitro* and *in vivo*. Immunosuppression is also an important risk factor for extrapulmonary tuberculosis. Gupta and Gupta discuss novel therapeutic approaches against female genital tuberculosis, representing one of the most perilous forms of extrapulmonary tuberculosis, and suggest that nuclear receptors could be major new therapeutic targets and/or diagnostic biomarkers.

An additional interesting approach, targeting local microbiota, has been described by Zhao et al. who report the use of *Clostridium butyricum*, a common human and animal gut commensal bacterium often used as a probiotic, as a possible treatment for *Salmonella enteriditis* infection. Here, *C. butyricum* attenuates inflammation and epithelial barrier damage, alters intestinal microbial composition, and increases the diversity of bacterial communities in the intestine of Salmonella infected chickens.

In conclusion, novel and heterogeneous therapeutic approaches to reduce the global burden of antimicrobial resistance have been proposed and discussed in this special issue. Based upon these studies, we suggest that a combination of both host- and pathogen- directed therapeutic approaches may represent a valuable and exploitable strategy, over single therapies, to i) control multidrug resistant infections, ii) minimize the risk of emergence of drug resistance and iii) reduce the time of therapy. This would, in turn, help reduce patient management costs in low- and middle-income countries where the social and economic impact of MDR burden has dramatic consequences.

## Author Contributions

All authors listed have made a substantial, direct, and intellectual contribution to the work and approved it for publication.

## Funding

This work was supported by the Italian Cystic Fibrosis Research Foundation (FFC#19/2019).

## Conflict of Interest

The authors declare that the research was conducted in the absence of any commercial or financial relationships that could be construed as a potential conflict of interest.
